# Fenofibrate mitigates microglial activation by reprogramming lipid metabolism and inhibiting ferroptosis

**DOI:** 10.1007/s11010-026-05544-8

**Published:** 2026-04-28

**Authors:** Ahmet Taskesen, Ceyhan Hacioglu, Sibel Tuncer, Guven Kilic, Cengiz Tuncer

**Affiliations:** 1 Faculty of Medicine, Department of Neurosurgery, Düzce University, Düzce, Turkey; 2 Faculty of Medicine, Department of Medical Biochemistry, Düzce University, Düzce, Turkey; 3 Faculty of Science, Department of Chemistry, Düzce University, Düzce, Turkey

**Keywords:** Microglia, Fenofibrate, Lipid metabolism, Ferroptosis, Neuroinflammation, Oxidative stress

## Abstract

Microglial activation is a central mediator of neuroinflammatory and neurodegenerative processes. Growing evidence indicates that dysregulated lipid metabolism and ferroptosis drive microglial dysfunction, yet pharmacological interventions targeting these interconnected pathways remain scarce. Fenofibrate is well-documented for its anti-inflammatory and antioxidant effects; nevertheless, its influence on microglial lipid metabolism and ferroptotic signaling remains unexplored. The present study was designed to examine the effects of fenofibrate on lipid remodeling, oxidative stress, and ferroptosis in human HMC3 microglia activated with lipopolysaccharide (LPS) and interferon-γ (IFN-γ). HMC3 cells were pre-treated with fenofibrate followed by inflammatory activation. Cell viability, cytokine secretion, oxidative stress, lipid droplet (LD) accumulation, and ferroptosis-associated markers were analyzed by ELISA, fluorescence imaging, qRT-PCR, and Western blotting. Fenofibrate decreased the production of TNF-α, IL-1β, and IL-6, restored mitochondrial membrane potential, and suppressed ROS and malondialdehyde (MDA) generation while increasing intracellular glutathione (GSH). The treatment markedly decreased LD accumulation by downregulating the lipid metabolism–related enzymes PLIN2, DGAT1, and GPAT4. Functional assays demonstrated enhanced fatty acid oxidation and restored lipolysis, directly confirming reprogrammed lipid catabolism. Moreover, fenofibrate attenuated ferroptotic stress, evidenced by reduced intracellular Fe²⁺ levels, decreased ACSL4 expression, and significant increase of the ferroptosis-protective enzyme GPX4. These molecular changes were accompanied by improved cell survival and decreased oxidative damage, suggesting that fenofibrate may partially modulate metabolic and and redox balance in activated microglia under in vitro conditions. In conclusion, these findings suggest that fenofibrate may exert protective effects by modulating lipid metabolism and suppressing ferroptosis-related pathways in activated microglia.

**Supplementary Information:**

The online version contains supplementary material available at 10.1007/s11010-026-05544-8.

## Introduction

Microglia, the principal immune effector cells of the central nervous system (CNS), serve as vigilant sentinels that continuously monitor the neural environment to maintain homeostasis and rapidly respond to pathological stimuli such as injury or infection [1]. In their resting or surveillant state, these cells exhibit a ramified morphology and play vital roles in maintaining neural integrity, including synaptic pruning, neurogenesis, and the secretion of neurotrophic factors that support neuronal survival [2]. However, under neurodegenerative conditions, microglia undergo a phenotypic transformation into a chronically activated state characterized by enhanced proliferation and the persistent secretion of pro-inflammatory mediators such as interleukin-6 (IL-6), interleukin-1β (IL-1β), and tumor necrosis factor-α (TNF-α) [3]. This prolonged inflammatory activation promotes oxidative stress and apoptotic signaling, thereby exacerbating neuronal injury and contributing to the progression of neurodegenerative pathology [4]. Although microglial activation exists along a dynamic and heterogeneous spectrum, it is commonly simplified into two functional phenotypes: the classically activated, pro-inflammatory M1 state and the alternatively activated, anti-inflammatory M2 state [5]. Activation toward the M1 phenotype—typically triggered by agents such as lipopolysaccharide (LPS) or interferon-γ (IFN-γ)—leads to the production of neurotoxic factors including nitric oxide (NO) via inducible nitric oxide synthase (iNOS), prostaglandin E2 (PGE2), and the cytokines IL-6 and IL-1β [6]. In contrast, M2-polarized microglia exhibit an anti-inflammatory profile characterized by the secretion of cytokines such as interleukin-10 (IL-10) and interleukin-4 (IL-4), which promote tissue repair, debris clearance, and resolution of inflammation [7]. This remarkable functional adaptability highlights the therapeutic potential of targeting microglial metabolic and lipid-regulatory pathways to modulate their polarization toward neuroprotective states in the context of neurodegenerative disease.

Parallel to inflammatory signaling, lipid metabolism serves as a cornerstone of cellular integrity, fulfilling roles that extend far beyond structural composition to include energy storage and sophisticated signal transduction [8]. A growing body of research underscores that disruptions in lipid handling within microglia are intimately linked to neurodegenerative pathogenesis by directly modulating immune responses [9]. Recent evidence also highlights a subset of microglia characterized by excessive lipid droplet (LD) accumulation, which exhibit reduced phagocytic efficiency, heightened oxidative stress, and enhanced secretion of pro-inflammatory mediators [10]. Such LD–rich microglia appear to emerge in response to chronic inflammation and may represent a dysfunctional state contributing to neurodegenerative progression [11]. The accumulation of specific lipid peroxidation products can induce microglial hyperactivation and the subsequent release of cytokines like IL-1β and TNF-α, creating a vicious cycle of inflammation and metabolic dysfunction [12]. Therefore, the intricate crosstalk between lipid metabolism pathways, and the production of specific pro-inflammatory cytokines represents an underexplored area of research with significant therapeutic implications.

The conceptual framework of ferroptosis, an iron-catalyzed mode of regulated cell death defined by the catastrophic oxidation of polyunsaturated fatty acids (PUFAs) within membrane lipids, has revealed a novel and critical nexus with aberrant lipid metabolism and microglial activation [13]. The ferroptotic process is initiated by a failure in the glutathione-dependent antioxidant defense mediated by glutathione peroxidase 4 (GPX4), resulting in the uncontrolled accrual of lethal lipid peroxides [14]. A growing body of research indicates that the pro-inflammatory polarization of microglia significantly heightens their susceptibility to undergoing ferroptosis [15]. The high metabolic demand and lipid remodeling associated with microglial activation can deplete key antioxidants like glutathione, thereby creating a permissive environment for lipid peroxidation [16]. Notably, the release of pro-inflammatory cytokines can further sensitize neighboring cells to ferroptotic damage, creating a feed-forward cycle of neuroinflammation and neuronal loss [17]. Emerging evidence indicates that the ferroptosis pathway is activated in microglia during neuroinflammation, contributing to their dysfunctional, hyperactivated state and the release of damaging lipid mediators, even in the absence of outright cell death [15]. Thus, elucidating the precise role of ferroptosis in microglial dysfunction and how lipid metabolism-regulating pathways modulate this cell death pathway, may represent a critical step in understanding neurodegenerative disease pathogenesis and identifying new therapeutic targets.

The pivotal involvement of lipid metabolism in directing microglial activation and determining susceptibility to ferroptosis positions the correction of such metabolic imbalances as a rational therapeutic objective. In this context, the pharmacological stimulation of peroxisome proliferator-activated receptor-α (PPAR-α) has garnered considerable attention as a promising intervention [18]. Fenofibrate, a well-established PPAR-α agonist clinically employed for managing dyslipidemia, is now being investigated for its neuroprotective potential, which stems from its two-pronged ability to potentiate fatty acid oxidation while concurrently attenuating neuroinflammatory pathways [19]. By activating PPAR-α–mediated gene transcription, fenofibrate augments mitochondrial β-oxidation, clears intracellular lipid droplets, and suppresses the expression of IL-1β, IL-6, and TNF-α [20]. Furthermore, the drug’s ability to enhance endogenous antioxidant mechanisms contributes to the suppression of reactive oxygen species (ROS) generation and the restoration of cellular redox homeostasis [21]. Together, these actions posit fenofibrate as a compelling candidate for ameliorating microglial dysfunction and associated neuronal injury driven by metabolic perturbations. Therefore, this study aimed to elucidate the influence of fenofibrate on microglial activation dynamics, lipid metabolic balance, and ferroptotic signaling pathways, with a particular focus on determining whether the compound preserves cellular integrity under conditions of inflammatory stress.

## Materials and methods

### Cell culture

All investigations in this study were conducted using the immortalized human microglial clone 3 (HMC3; ATCC^®^ CRL-3304) cell line. Routine cell culture was maintained in Minimum Essential Medium (MEM) supplemented with 10% heat-inactivated fetal bovine serum (FBS), 2 mM L-glutamine, and a 1% antibiotic solution of penicillin/streptomycin. Cells were cultivated under standard conditions in a humidified incubator providing a 5% CO₂ atmosphere at 37 °C. A standard subculturing protocol was implemented upon cells reaching approximately 80% confluency, utilizing a 0.25% trypsin-EDTA solution for enzymatic dissociation.

### Cell treatment and microglial activation

**Fig. 1:**
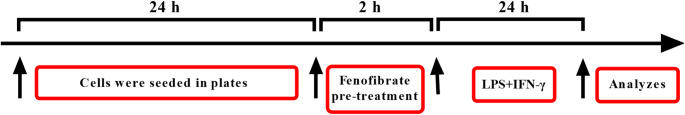
Schematic representation of the experimental treatment protocol. Cells were initially cultured overnight to achieve adequate adherence and confluence. Following this stabilization period, the cultures were pre-treated with fenofibrate for a duration of 2 h. Subsequently, the cells were exposed to a pro-inflammatory stimulus consisting of a combination of LPS and IFN-γ for 24 h to induce activation

To establish a pro-inflammatory model, HMC3 cells were seeded into 96-well plates at a density of 1 × 10⁴ cells per well and allowed to adhere for 24 h. Fenofibrate (Sigma-Aldrich, F6020) was dissolved in dimethyl sulfoxide (DMSO) to prepare a 100 mM stock solution, which was subsequently diluted in culture medium to obtain final concentrations of 0, 1, 5, 10, 20, 50, and 100 µM. The final DMSO concentration was adjusted to 0.1% in all treatment groups to ensure consistency. After a 2-h pre-treatment with fenofibrate, microglial activation was stimulated by the combined application of 100 ng/mL lipopolysaccharide (LPS) and 10 ng/mL interferon-γ (IFN-γ) for 24 h, as illustrated in Fig. 1.

### Cell viability assay

Cellular viability in response to fenofibrate treatment was assessed quantitatively through a colorimetric Cell Counting Kit-8 (CCK-8; E-CK-A362) assay. Following the 24-hour experimental incubation period, the CCK-8 reagent was introduced directly into each well of the culture plate. The plate was subsequently returned to a 37 °C incubator for a specified duration, adhering strictly to the manufacturer’s protocol. The metabolic activity of the cells, which correlates directly with viability, was then determined by measuring the absorbance of the resulting colored solution at a primary wavelength of 450 nm utilizing a BioTek microplate reader. The percentage of viable cells for each treatment condition was calculated based on the following relationship: Viability (%) = [(Absorbance_treated - Absorbance_blank) / (Absorbance_control - Absorbance_blank)] × 100.

### Cell proliferation

Cellular proliferation rates were evaluated via a colorimetric bromodeoxyuridine (BrdU) incorporation assay (Sigma-Aldrich, #2750). This method detects the integration of BrdU, a thymidine analog, into nascent DNA strands during the S-phase of the cell cycle. After the experimental treatments, cells were labeled with BrdU, fixed, and subsequently processed in accordance with the supplied protocol. The degree of BrdU incorporation was determined by measuring the absorbance at 450 nm.

### Biochemical analyses

HMC3 microglial cells were distributed into culture plates at a seeding density of 10,000 cells per well and systematically assigned to one of three experimental groups: a control group (I), an inflammatory challenge group exposed to LPS/IFN-γ (II), and an intervention group that received fenofibrate pre-treatment before LPS/IFN-γ stimulation (III). Upon completion of the designated treatments, the cellular monolayers were rinsed with ice-cold phosphate-buffered saline (PBS, pH 7.4) to remove extracellular components. Cells were then detached from the substrate using a trypsin-EDTA solution and collected by centrifugation at 1000 × g for 5 min at 4 °C. The harvested cell pellets underwent multiple washing cycles with chilled PBS to ensure complete removal of medium contaminants. Subsequent protein extraction was achieved by resuspending the cell pellets in RIPA lysis buffer (Santa Cruz) and incubating the suspensions at 4 °C for 30 min with constant agitation to facilitate complete lysis. The resulting lysates were clarified by high-speed centrifugation at 16,000 × g for 10 min at 4 °C to pellet insoluble cellular debris. Protein quantification was performed on the clarified supernatants using a bicinchoninic acid (BCA) protein assay kit (Elabscience, #E-BC-K318-M), followed by normalization of all samples to a consistent concentration of 1.0 mg/mL in preparation for subsequent analytical procedures, specifically enzyme-linked immunosorbent assays (ELISA).

The concentrations of ferrous iron (Fe²⁺), IL-1β, TNF-α, IL-6, malondialdehyde (MDA), and glutathione (GSH) in the cell lysates were quantified using specific commercial ELISA kits (MAK025, RAB0273, RAB0476, EZHIL6, MAK085, MBS727656, respectively). According to the standard protocol, the prepared lysates were applied to the antibody-coated microplate. The plate was incubated once more to facilitate the formation of the antibody-antigen complex, followed by another wash to eliminate any unbound detection antibody. The intensity of the resultant color, measured with a microplate reader, was directly proportional to the concentration of the target cytokine in the original sample.

### Detection of neutral lipid accumulation

The visualization of intracellular LD accumulation was performed employing HCS LipidTOX™ Deep Red Neutral Lipid Stain (Invitrogen, H34477). HMC3 microglial cells were initially seeded into confocal-compatible dishes at a density of 10,000 cells per well and granted a 24-hour period for complete adhesion. Subsequently, the standard culture medium was aspirated and replaced with a specialized staining solution, comprising 50 µM of the LipidTOX™ reagent dissolved in growth medium that had been pre-supplemented with the designated concentrations of fenofibrate. Cellular incubation with this staining solution proceeded for 30 min under standard culture conditions (37 °C, 5% CO₂), protected from light to prevent fluorophore degradation. Following this incubation, the staining solution was removed, and any residual, unincorporated dye was thoroughly eliminated through multiple washes with Dulbecco’s Phosphate-Buffered Saline (DPBS). Immediately preceding microscopic analysis, the DPBS was replaced with fresh RPMI medium to ensure optimal imaging conditions. Confocal imaging was subsequently carried out using an Olympus laser scanning microscope (Tokyo, Japan), with optical configurations precisely calibrated to the deep red emission spectrum of the dye for specific signal capture.

### Fatty Acid Oxidation (FAO) Assay

The rate of mitochondrial β-oxidation was measured using a commercially available Fatty Acid Oxidation Assay Kit (E-BC-K784-M). Briefly, HMC3 cells in the various treatment groups were incubated with a palmitate-BSA conjugate. The oxidation reaction generated NADH, which was coupled to a color-changing reporter. Absorbance was measured over 60 min using a microplate reader. The rate of NADH generation was calculated and normalized to protein content, reflecting FAO activity.

### Lipolysis assay

To quantify the breakdown of triglycerides stored within LDs, the release of free glycerol into the culture medium was measured using a Glycerol Colorimetric Assay Kit (E-BC-K340-M). After treatments, cell culture supernatants were collected, and glycerol content was determined according to the manufacturer’s protocol by measuring absorbance at 510 nm.

### Western blot analysis

For Western blot analysis, cellular protein extracts were obtained by lysing cells in RIPA buffer (Thermo Scientific, #89900). Following quantification of protein content, aliquots containing 20 µg of protein from each sample were subjected to electrophoretic separation on 10% SDS-polyacrylamide gels. The resolved proteins were then transferred to polyvinylidene fluoride (PVDF) membranes via electroblotting. Membrane blocking was carried out for 2 h at ambient temperature using a solution of 5% bovine serum albumin (BSA) in Tris-buffered saline containing 0.05% Tween-20 (TBST). Primary antibody incubations were conducted overnight at 4 °C with specific antibodies targeting the following proteins: TNF-α (diluted 1:1000; Thermo Fisher, PA1-40281), CD68 (diluted 1 µg/mL; Thermo Fisher, 14-0688-82), IL-1β (diluted 1 µg/mL; Thermo Fisher, P420B), PLIN2 (diluted 1:1000; Cell Signaling Technology, #45535), glycerol-3-phosphate acyltransferase 4 (GPAT4) (diluted 1:1000; Cell Signaling Technology, #66933), diacylglycerol O-acyltransferase 1 (DGAT1) (diluted 1:1000; Thermo Fisher, PA5-117074), and β-actin (diluted 1:5000, Sigma-Aldrich, A5441). After thorough washing to remove unbound antibodies, membranes were incubated with species-appropriate horseradish peroxidase (HRP)-conjugated secondary antibodies. Immunoreactive bands were detected using an electrochemiluminescence (ECL) substrate system (Thermo Scientific, #34579), and the resulting band intensities were quantified through densitometric analysis with ImageJ software, normalized against the corresponding β-actin signal.

### Quantitative real-time polymerase chain reaction (qRT-PCR)

Total RNA was extracted using TRIzol reagent (Invitrogen) and subsequently treated with DNase I to eliminate residual genomic DNA contamination. The purity and integrity of RNA samples were verified by confirming an A260/A280 ratio within the range of 1.8 to 2.0. Complementary DNA (cDNA) was synthesized from 1 µg of total RNA using the SuperScript™ IV Reverse Transcriptase kit, following the manufacturer’s protocol. Quantitative real-time PCR (qRT-PCR) was conducted in triplicate using Power SYBR™ Green Master Mix on a StepOnePlus Real-Time PCR System. Relative gene expression levels were determined using the 2^⁻ΔΔCt^ method, with β-actin serving as the internal normalization control. The nucleotide sequences of the primers used were: GPX4: Forward 5′-ACC GTA AGG ACT ACT CGT GAG-3′ - Reverse 5′-AGG CCT ATG CCG GAA CAT CC-3′; ACSL4: Forward 5′-CAG CCA AAG GGT CAT GCG C-3′ - Reverse 5′-AGG CCT GCC ATA AAT GCA-3′; iNOS: Forward 5′-GGT CGC AGA TCA ACA ATA CAA GC-3′ - Reverse 5′-GCG GAT AGG TTG ATG ACA C-3′; CD86: Forward 5′- GGC ATA GTC TCA AAA CCA CAG-3′ - Reverse 5′- GGC ATA CGC TGG GCT TCA GC-3′; Arginase-1 (Arg1): Forward 5′- CGC CTA GAC TAA GTC CTT AGG-3′ - Reverse 5′- AGG AGC CGT CGT AAG GGA CAG-3′; β-actin: Forward 5′-CGA CGG CTA ACG AGA TTA GGG-3′ - Reverse 5′-AGG CCT TTG CTC CGG GAA CGC-3′.

### Measurement of intracellular superoxide anions

Intracellular superoxide generation was evaluated using the fluorescent probe dihydroethidium (DHE). Following treatment, cells were rinsed with phosphate-buffered saline (PBS) and incubated with 10 µM DHE diluted in serum-free medium for 30 min at 37 °C under dark conditions to prevent photobleaching. After incubation, unbound probe was removed by additional PBS washes, and fluorescence signals were visualized using confocal laser scanning microscopy. Quantitative analysis of mean fluorescence intensity—reflecting intracellular superoxide levels—was performed using ImageJ.

### Assessment of intracellular iron accumulation

Intracellular iron levels were evaluated cytochemically using Prussian Blue staining. Cells were seeded on sterile glass coverslips in 12-well plates at a density of 1 × 10⁵ cells per well and subjected to the experimental treatments. To investigate the contribution of ferroptotic mechanisms, a separate set of experiments included a 2-h pre-treatment with the iron chelator desferrioxamine (DFO; 10 µM; Sigma-Aldrich D9533) prior to fenofibrate application, as per established protocols [22]. After fixation with 4% paraformaldehyde, cells were treated with a freshly prepared mixture of 2% potassium ferrocyanide and 2% hydrochloric acid (1:1 ratio) for 30 min. Cell nuclei were counterstained with Nuclear Fast Red, and images were acquired using bright-field microscopy.

### Immunofluorescence analysis

The subcellular distribution of ACSL4 and GPX4 proteins was examined through immunofluorescence analysis. Cells were initially fixed in 4% paraformaldehyde to preserve structural integrity, followed by permeabilization with 0.2% Triton X-100 to facilitate antibody access. Non-specific binding sites were subsequently blocked using 5% bovine serum albumin (BSA). Thereafter, the samples were incubated overnight at 4 °C with primary antibodies targeting ACSL4 (1:200 dilution, Thermo Fisher, PA5-27137) and GPX4 (1:500 dilution, Thermo Fisher, PA5-102521). Following extensive washing to remove unbound antibodies, fluorophore-labeled secondary antibodies (1:1000 dilution) were applied for 60 min under light-protected conditions. Confocal laser scanning microscopy was employed to visualize the fluorescent signals, and quantitative assessment of fluorescence intensity was performed using ImageJ software to determine the relative localization and expression patterns of the target proteins.

### PPAR-α antagonist experiment

To investigate whether the protective effects of fenofibrate are mediated through PPAR-α signaling, additional experiments were designed using the selective PPAR-α antagonist GW6471. HMC3 microglial cells were pretreated with GW6471 (10 µM) for 1 h prior to fenofibrate exposure, followed by LPS/IFN-γ stimulation as described above. After 24 h of treatment, cell viability was assessed using the CCK-8 assay according to the manufacturer’s instructions. In parallel, inflammatory cytokine levels (TNF-α, IL-1β, IL-6) were quantified using ELISA kits. Oxidative stress parameters, including MDA and GSH, were measured to evaluate lipid peroxidation and antioxidant status. Ferroptosis-related markers, including intracellular Fe²⁺ levels, GPX4, and ACSL4 expression, were also analyzed using ELISA-based methods.

### Statistical analysis

All experiments included a minimum of seven independent biological replicates. Data are expressed as the mean ± standard error of the mean (SEM). Comparisons between two groups were analyzed using an unpaired Student’s t-test. For comparisons across multiple groups, one-way or two-way analysis of variance (ANOVA) was employed, followed by Tukey’s post-hoc test for multiple comparisons. A probability value (p) of less than 0.05 was considered statistically significant. All statistical analyses were performed using GraphPad Prism software, version 8.0.

## Results

### Fenofibrate exhibits cytotoxicity and anti-proliferative effects on HMC3 microglia at high concentrations

**Fig. 2:**
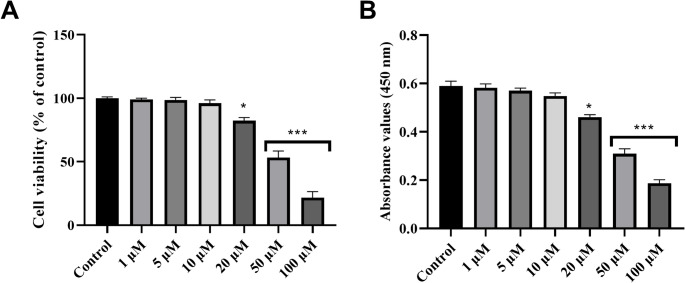
Fenofibrate reduces HMC3 microglial cell viability and proliferation in a concentration-dependent manner. **A** Cell viability was assessed using the CCK-8 assay in HMC3 cells treated with increasing concentrations of fenofibrate (0–100 µM) for 24 h. **B** Cell proliferation was evaluated using the BrdU incorporation assay under the same treatment conditions. Data are presented as mean percentage of control ± SEM (*n* = 7 independent experiments). Statistical comparisons between multiple groups were performed using one-way ANOVA followed by Tukey’s post-hoc test. *p < 0.05, and ***p < 0.0001 compared to the control group

To establish a non-cytotoxic concentration for subsequent investigations, HMC3 microglial cells were exposed to escalating concentrations of fenofibrate (0–100 µM). Cellular viability and proliferative capacity were robustly quantified through colorimetric CCK-8 assay, and BrdU incorporation assay (Fig. 2). Comprehensive analysis revealed that fenofibrate concentrations up to 10 µM (specifically 0, 1, 5, and 10 µM) exhibited no significant cytotoxicity or anti-proliferative effects relative to vehicle-treated controls (*p* > 0.05). In contrast, a marked concentration-dependent reduction in both viability and proliferation indices was observed commencing at 20 µM, establishing this threshold as the onset of significant cellular toxicity.

Specifically, fenofibrate at 25, 50, and 100 µM significantly reduced cell viability by 14.2%, 45.4%, and 88.2%, respectively (Fig. 2A). Based on these dose-response data, the half-maximal inhibitory concentration (IC50) of fenofibrate in HMC3 cells was calculated to be 58.3 µM. In parallel with the viability assays, the proliferation rate of activated HMC3 cells was also suppressed at these higher concentrations. The BrdU assay revealed that fenofibrate at 25, 50, and 100 µM decreased cellular proliferation by 11.8%, 42.5%, and 90.4%, respectively (Fig. 2B). Consequently, a concentration of 20 µM was selected for subsequent experiments to evaluate the regulatory effects of fenofibrate on HMC3 cells.

### Fenofibrate attenuates LPS/IFN-γ-induced microglial activation by suppressing pro-inflammatory mediators in HMC3 cells

To establish a pro-inflammatory phenotype in vitro, HMC3 human microglial cells were subjected to a 2-h pre-treatment with fenofibrate prior to exposure to a co-stimulatory cocktail containing 100 ng/mL LPS and 10 ng/mL IFN-γ for a 24-h period. The effectiveness of this inflammatory induction was assessed by evaluating the expression of canonical pro-inflammatory mediators. Western blot analyses demonstrated a pronounced upregulation of TNF-α, CD68—an established marker of microglial activation—and IL-1β in cells exposed to LPS and IFN-γ relative to untreated controls.

**Fig. 3:**
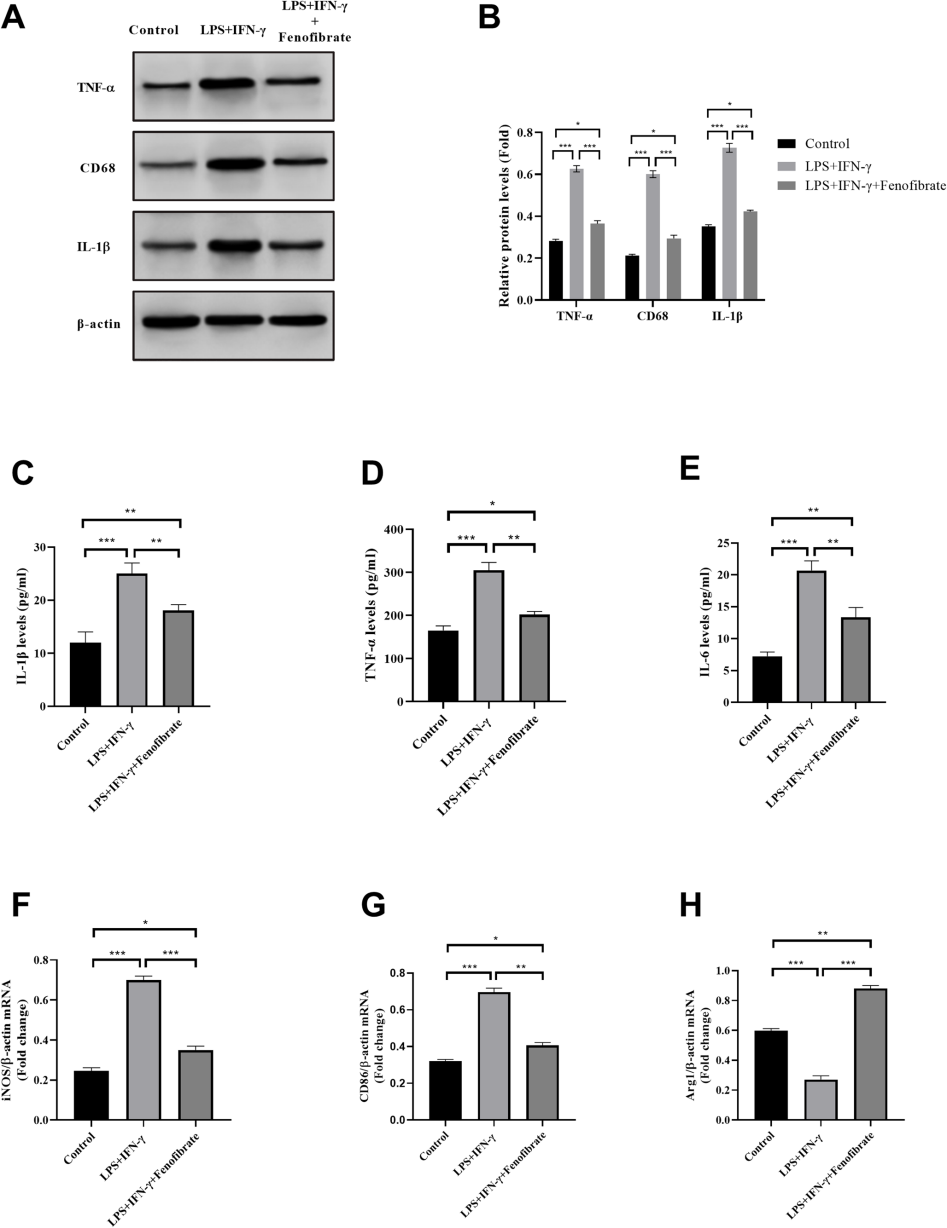
Fenofibrate attenuates LPS/IFN-γ-induced pro-inflammatory marker expression in activated HMC3 microglial cells. **A** Representative Western blot images showing protein expression levels of TNF-α, CD68, and IL-1β. **B** Densitometric quantification of relative protein levels normalized to β-actin. ELISA analysis of secreted **C** IL-1β, **D** TNF-α, and **E** IL-6 levels in cell culture supernatants. qRT-PCR analysis of mRNA expression levels of **F** iNOS and **G** CD86 as M1 polarization markers and **H** Arg1 as an M2-associated marker. Cells were pre-treated with 20 µM fenofibrate for 2 h followed by activation with LPS (100 ng/mL) and IFN-γ (10 ng/mL) for 24 h. Data are presented as mean ± SEM (*n* = 7 independent experiments). Statistical comparisons between multiple groups were performed using one-way ANOVA followed by Tukey’s post-hoc test. Statistical comparisons between protein analyses were performed using the two-way ANOVA test. *p < 0.05, **p < 0.001, and ***p < 0.0001

Quantitative assessment, as illustrated in Fig. 3, revealed that LPS + IFN-γ stimulation led to a significant elevation in all three proteins. TNF-α expression increased approximately 1.5-fold, CD68 levels rose by ~ 1.8-fold, and IL-1β exhibited a 1.2-fold enhancement compared to the control group (all *p* < 0.0001; Fig. 3B). Representative Western blot images (Fig. 3A) indicate these robust protein expression changes in the activated cells. Post-treatment with 20 µM fenofibrate substantially mitigated this inflammatory response. Protein quantification indicated that fenofibrate reduced TNF-α, CD68, and IL-1β expression by 43.7%, 35.1%, and 41.9%, respectively, compared to the LPS + IFN-γ group (*p* < 0.0001), indicating a significant anti-inflammatory effect of fenofibrate in this microglial model.

In agreement with the Western blot findings, ELISA assays confirmed an elevation of pro-inflammatory cytokines in HMC3 cells following stimulation with LPS and IFN-γ. Quantitative measurements indicated that IL-1β, TNF-α, and IL-6 levels were significantly elevated in the activated cells relative to untreated controls, as shown in Fig. 3C and D. Specifically, IL-1β levels were elevated approximately 2.5-fold, TNF-α showed an approximate 1.8-fold increase, and IL-6 exhibited the most pronounced upregulation of approximately 3.1-fold (*p* < 0.0001). In contrast, the administration of 20 µM fenofibrate significantly attenuated this inflammatory response. ELISA analysis confirmed that fenofibrate treatment reduced the secreted levels of IL-1β, TNF-α, and IL-6 by 39.1%, 32.5%, and 42.6%, respectively, compared to the LPS + IFN-γ group. These data collectively confirm that the LPS + IFN-γ treatment protocol successfully induced a microglial activation in HMC3 cells, while also demonstrating the anti-inflammatory capacity of fenofibrate to suppress the secretion of these critical inflammatory cytokines.

To further characterize the phenotypic shift, we analyzed additional polarization markers (Fig. 3F, G and H). LPS/IFN-γ stimulation significantly increased mRNA expression of M1-associated genes (iNOS, CD86) while decreasing M2-associated genes (Arg1). Fenofibrate pre-treatment mitigated the upregulation of M1 markers and promoted a significant increase in Arg1 expression, suggesting a partial shift away from a pro-inflammatory phenotype.

### Fenofibrate reverses activated microglial lipid metabolic reprogramming by downregulating key lipid droplet-associated proteins

To elucidate the potential relationship between differential treatment response and lipid metabolic reprogramming in activated versus resting HMC3 microglia, we focused on key regulators of lipid uptake, storage, and utilization. Specifically, we investigated the expression of proteins and enzymes critical for lipid droplet (LD) dynamics: PLIN2, a regulator of LD lipolysis and degradation; GPAT4, which facilitates the formation of large LDs; and DGAT1, involved in the formation of smaller LDs.

**Fig. 4:**
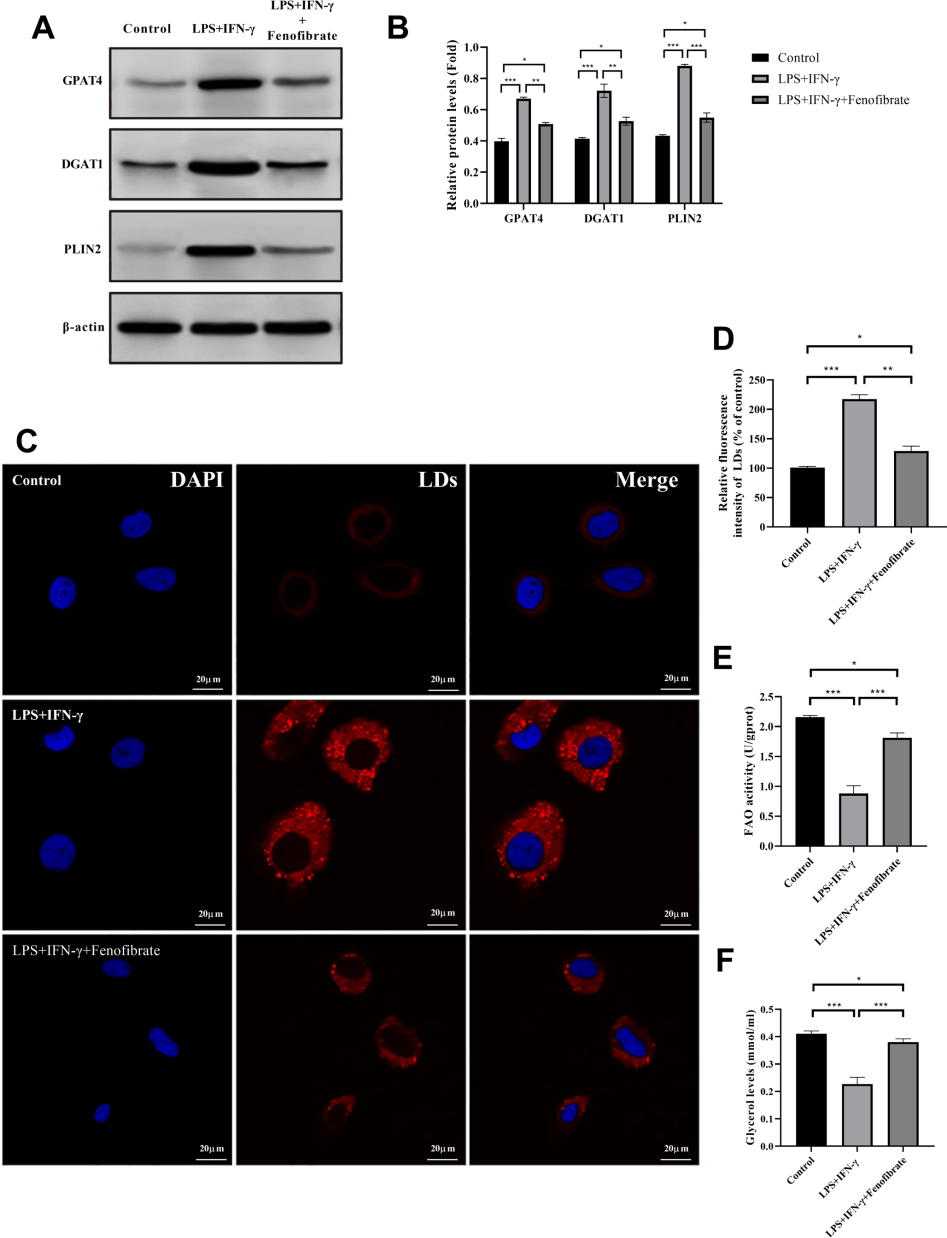
Fenofibrate reverses lipid metabolic reprogramming in activated HMC3 microglial cells. **A** Representative Western blot images showing protein expression levels of GPAT4, DGAT1, and PLIN2. **B** Densitometric quantification of relative protein levels normalized to β-actin. **C** Representative fluorescent images of lipid droplets stained with LipidTOX™ Deep Red Neutral Lipid Stain (scale bar: 20 μm). **D** Quantitative analysis of relative fluorescence intensity of lipid droplets. **E** FAO acitivity. **F** Glycerol levels. Cells were pre-treated with 20 µM fenofibrate for 2 h followed by activation with LPS (100 ng/mL) and IFN-γ (10 ng/mL) for 24 h. Data are presented as mean ± SEM (*n* = 7 independent experiments). Statistical comparisons between multiple groups were performed using one-way ANOVA followed by Tukey’s post-hoc test. Statistical comparisons between protein analyses were performed using the two-way ANOVA test. *p < 0.05, **p < 0.001, and ***p < 0.0001

Western blot and LD staining analyses were employed to quantitatively assess the protein expression levels of these metabolic markers. Our findings revealed that activated HMC3 cells exhibited a significant upregulation in the expression of GPAT4, DGAT1, and PLIN2 compared to their resting counterparts (Fig. 4A and B). Quantification demonstrated that GPAT4 and DGAT1 expression increased by 57.2% and 42.5%, respectively, while PLIN2 levels were augmented by 78.6%. Critically, LD staining revealed a concomitant and significant increase in LD density, providing visual confirmation of the protein upregulation data obtained by Western blot (Fig. 4C and D). This consistent increase in both the core regulatory proteins and their functional output in the form of LD accumulation suggests that enhanced lipid import, storage, and turnover mechanisms may contribute to the activated microglial phenotype.

Conversely, fenofibrate treatment led to a marked reduction in intracellular LDs in activated HMC3 cells (Fig. 4C and D). This observed decrease in LD accumulation was associated with a corresponding downregulation in the protein expression levels of GPAT4, DGAT1, and PLIN2 across treatment groups (Fig. 4A, B). Specifically, fenofibrate administration significantly reduced GPAT4, DGAT1, and PLIN2 expression by 27.5%, 32.1%, and 40.7%, respectively, compared to the activated HMC3 cells. These findings suggest that the reduction in LD content is associated with the downregulation of GPAT4, DGAT1, and PLIN2 expression following fenofibrate treatment.

Building on our initial observation that fenofibrate reduces LD accumulation, we sought to determine if this was due to enhanced lipid metabolism. We first assessed mitochondrial FAO and found that LPS/IFN-γ activation significantly suppressed the FAO rate compared to the non-activated control group (*p* < 0.0001; Fig. 4E). Fenofibrate treatment not only reversed this suppression but elevated the FAO rate to 1.3-fold above the activated control level (*p* < 0.0001), consistent with its role as a PPAR-α agonist.

Furthermore, we measured lipolytic activity by quantifying glycerol release. Activated HMC3 cells showed a 47% reduction in extracellular glycerol, indicating impaired lipolysis (Fig. 4F; *p* < 0.0001). Fenofibrate pre-treatment restored glycerol release to levels comparable to the non-activated control, demonstrating its ability to promote the breakdown of stored lipids.

### Fenofibrate suppresses lipid peroxidation and ferroptosis by regulating intracellular ROS in activated microglia

**Fig. 5:**
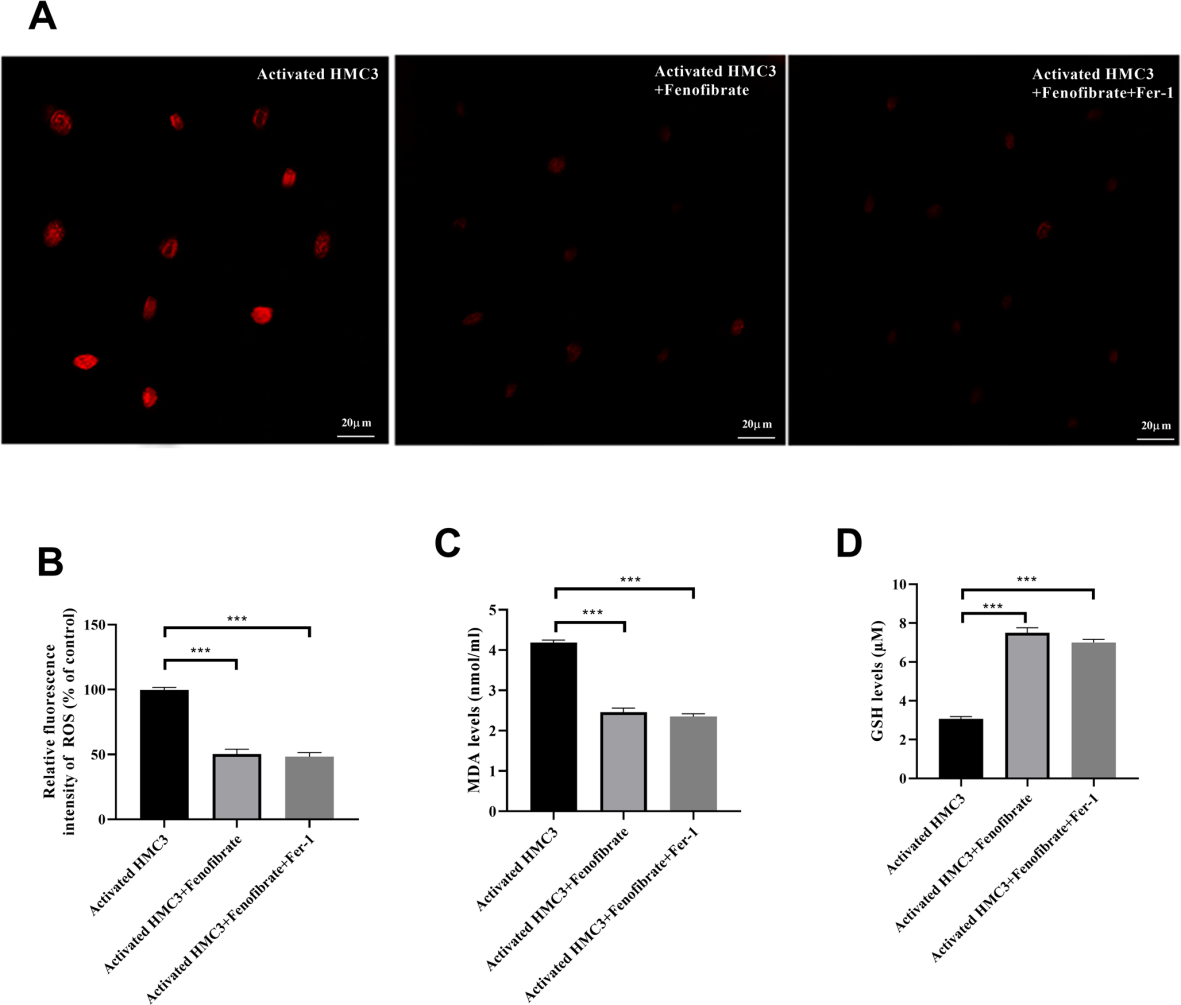
Fenofibrate mitigates oxidative stress in activated HMC3 microglial cells. **A** Representative fluorescent images of intracellular ROS detection using DHE staining (scale bar: 20 μm). **B** Quantitative analysis of relative DHE fluorescence intensity. ELISA analysis of **C** MDA levels and **D** GSH concentrations. Cells were pre-treated with 20 µM fenofibrate for 2 h followed by activation with LPS (100 ng/mL) and IFN-γ (10 ng/mL) for 24 h. Data are presented as mean ± SEM (*n* = 7 independent experiments). Statistical comparisons between multiple groups were performed using one-way ANOVA followed by Tukey’s post-hoc test. ***p < 0.0001

Intracellular ROS production was assessed using dihydroethidium (DHE) to specifically detect superoxide and hydroxyl radicals. Activated HMC3 cells exhibited a significant increase in ROS levels, which was substantially attenuated by fenofibrate treatment. Quantitative analysis revealed that fenofibrate reduced intracellular ROS by approximately 48.3% relative to the activated, untreated group (*p* < 0.0001; Fig. 5A and B). In addition, fenofibrate elicited a pronounced antioxidant effect, reflected by a marked reduction in malondialdehyde (MDA), a lipid peroxidation marker, alongside a significant elevation in intracellular glutathione (GSH) levels. Specifically, MDA content decreased by 52.7% (*p* < 0.0001, Fig. 5C), whereas GSH levels increased by 65.1% (*p* < 0.0001, Fig. 5E) compared with activated control cells.

To investigate the contribution of ferroptosis to fenofibrate’s protective effects, HMC3 cells were pre-incubated with the ferroptosis-specific inhibitor ferrostatin-1 (Fer-1, 10 µM) for 2 h prior to fenofibrate treatment. This intervention effectively normalized intracellular ROS and MDA levels while restoring GSH content, producing outcomes comparable to those observed in fenofibrate-treated activated microglia. Collectively, these findings indicate that fenofibrate treatment is associated with reduced ROS accumulation, decreased lipid peroxidation, and increased antioxidant defenses in activated HMC3 cells. Importantly, these data suggest that fenofibrate mitigates cellular damage in activated microglia through modulation of a lipid peroxidation–dependent ferroptotic pathway.

**Fig. 6:**
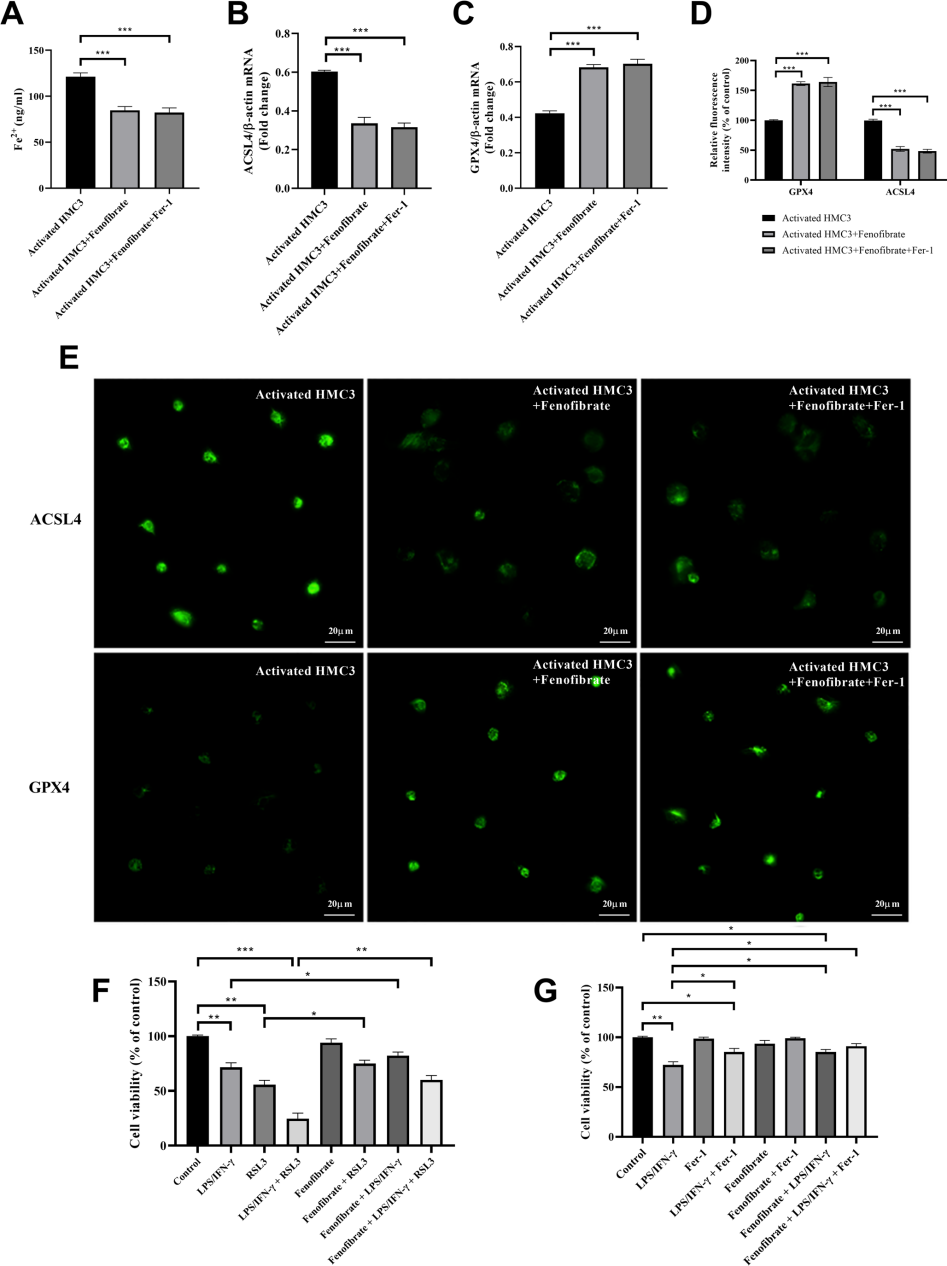
Fenofibrate modulates ferroptotic signaling in activated HMC3 microglial cells. **A** Intracellular Fe²⁺ levels measured by ELISA. **B** Relative ACSL4 and **C** GPX4 mRNA expression levels determined by qRT-PCR. **D** Quantitative analysis of immunofluorescence intensity and **E** representative immunofluorescence images of ACSL4 and GPX4 protein expression (scale bar: 20 μm). Cells were pre-treated with 20 µM fenofibrate with 2-hour Fer-1 (10 µM) pre-treatment, followed by activation with LPS (100 ng/mL) and IFN-γ (10 ng/mL) for 24 h. **F** Fenofibrate protects against RSL3-induced ferroptotic cell death. **G** Ferroptosis inhibition rescues cell viability in activated microglia. Cell viability was assessed by CCK-8 assay after 24 h treatment with indicated combinations of fenofibrate (20 µM), LPS/IFN-γ, and the GPX4 inhibitor RSL3 (1 µM), and the ferroptosis inhibitor Fer-1 (10 µM). Data are presented as mean ± SEM (*n* = 7 independent experiments). Statistical comparisons between multiple groups were performed using one-way ANOVA followed by Tukey’s post-hoc test. Statistical comparisons between relative fluorescence intensity analyses were performed using the two-way ANOVA test. * *p* < 0.05, ** *p* < 0.001, and *** *p* < 0.0001

To further explore fenofibrate’s impact on ferroptotic signaling, intracellular Fe²⁺ concentrations and the expression of key ferroptosis-associated proteins were assessed. Activated HMC3 cells exhibited a significant increase in labile Fe²⁺, which was substantially reduced by fenofibrate treatment, showing a 42.5% decrease relative to activated controls (*p* < 0.0001; Fig. 6A). Pre-treatment with Fer-1 produced similar normalization of Fe²⁺ levels, corroborating the specific involvement of ferroptotic mechanisms.

Transcriptional regulation of the ferroptotic pathway was examined by quantifying GPX4 and ACSL4 mRNA levels using qRT-PCR. Fenofibrate significantly modulated the expression of these critical regulators in activated microglia. Specifically, GPX4, an anti-ferroptotic gene, was upregulated by approximately 63.8% (*p* < 0.0001; Fig. 6C), whereas ACSL4, a pro-ferroptotic gene, was downregulated to 55% of the level observed in activated controls (*p* < 0.0001; Fig. 6B). Pre-treatment with Fer-1 elicited analogous transcriptional changes, further confirming that fenofibrate exerts its protective effects through modulation of the ferroptotic pathway.

These transcriptional findings were further corroborated at the protein level by immunofluorescence analysis of the critical ferroptosis regulators GPX4 and ACSL4. Fenofibrate administration significantly upregulated the anti-ferroptotic protein GPX4 by 61.2% and, conversely, downregulated the pro-ferroptotic protein ACSL4 by 48.3% (*p* < 0.0001, Fig. 6D and E). In accordance with the biochemical and gene expression data, pre-treatment with Fer-1 yielded similar alterations in GPX4 and ACSL4 protein expression, mirroring the protective effects of fenofibrate. Collectively, these multi-level data collectively indicate that fenofibrate is associated with protective effects against microglial activation by modulating key ferroptotic mediators at both the transcriptional and translational levels, specifically through the inhibition of iron accumulation and lipid peroxidation via the upregulation of GPX4 and suppression of ACSL4.

To directly assess ferroptotic cell death, cells were co-treated with the GPX4 inhibitor RSL3 (1 µM) and fenofibrate for 24 h (Fig. 6F). Viability was assessed using CCK-8. Consistent with ferroptotic susceptibility, RSL3 alone significantly reduced viability to 55.3 ± 3.4% (*p* < 0.001 vs. control), confirming its role in inducing ferroptosis. Inflammatory activation alone (LPS/IFN-γ) slightly affected viability (73.5 ± 3.2%). However, the combination of LPS/IFN-γ and RSL3 synergistically exacerbated cell death, reducing viability to 28.6 ± 4.5% (*p* < 0.0001 vs. control), indicating heightened ferroptotic susceptibility in activated microglia. Notably, fenofibrate pre-treatment conferred robust protection against this combined insult. Viability in the Fenofibrate + LPS/IFN-γ + RSL3 group was 61.7 ± 3.6%, representing a significant rescue compared to the LPS/IFN-γ + RSL3 group (*p* < 0.001). Fenofibrate alone or in combination with LPS/IFN-γ had no adverse effect on viability (95.8 ± 2.9% and 85.2 ± 2.4%, respectively). Furthermore, fenofibrate also partially attenuated cell death induced by RSL3 alone (Fenofibrate + RSL3: 75.1 ± 3.0% vs. RSL3 alone: 55.3 ± 3.4%; *p* < 0.05).

Furthermore, to confirm that ferroptotic stress underlies the reduced viability in activated microglia, we employed the ferroptosis inhibitor Fer-1. As shown in Fig. 6G, Fer-1 significantly rescued the viability of LPS/IFN-γ-treated cells (87.2 ± 1.8% vs. 72.6 ± 2.5% in the LPS/IFN-γ group; *p* < 0.05). Fenofibrate pre-treatment showed a comparable restorative effect (96.2 ± 1.7%). Notably, the combination of fenofibrate and Fer-1 (97.1 ± 1.1%) did not yield a statistically significant additive benefit compared to fenofibrate alone (*p* > 0.05), confirming that both interventions converge on inhibiting the ferroptotic pathway to promote cell survival.

**Fig. 7:**
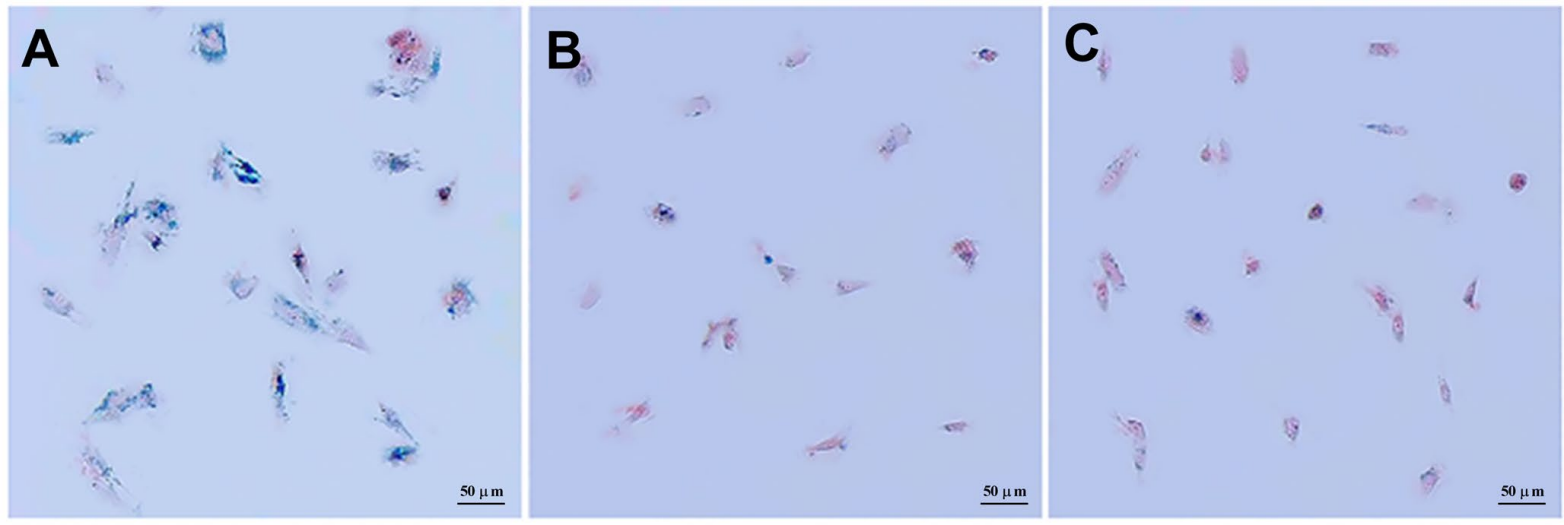
Fenofibrate-induced reduction of iron accumulation in activated HMC3 microglial cells is rescued by DFO. Representative images of Prussian Blue staining (blue deposits indicate iron accumulation) with Nuclear Fast Red counterstaining (pink, nuclei) in activated HMC3 cells. **A** Untreated activated control group, **B** Fenofibrate treated group (20 µM), and **C** Fenofibrate-treated HMC3 cells pre-treated with the iron chelator DFO (10 µM). Cells were pre-treated with 20 µM fenofibrate with 2-hour DFO (10 µM) pre-treatment, followed by activation with LPS (100 ng/mL) and IFN-γ (10 ng/mL) for 24 h. Scale bar = 50 μm

Prussian Blue staining was employed to provide cytochemical validation of the iron accumulation detected in our biochemical analysis (Fig. 7). The staining revealed a significant decrease in intracellular iron deposits upon fenofibrate treatment compared to the untreated activated control (Fig. 7A and B), a finding consistent with the quantitative Fe²⁺ levels. This fenofibrate-induced iron deposition was effectively abrogated when cells were pre-treated with the iron chelator DFO (Fig. 7C). The ability of DFO to prevent iron accumulation substantiates the critical involvement of iron overload in the activation of the ferroptosis pathway under these experimental conditions.

### PPAR-α antagonism attenuates the protective effects of fenofibrate

To determine whether the anti-inflammatory and anti-ferroptotic effects of fenofibrate are mediated through PPAR-α signaling, HMC3 microglial cells were pretreated with the selective PPAR-α antagonist GW6471 prior to fenofibrate exposure.

**Fig. 8:**
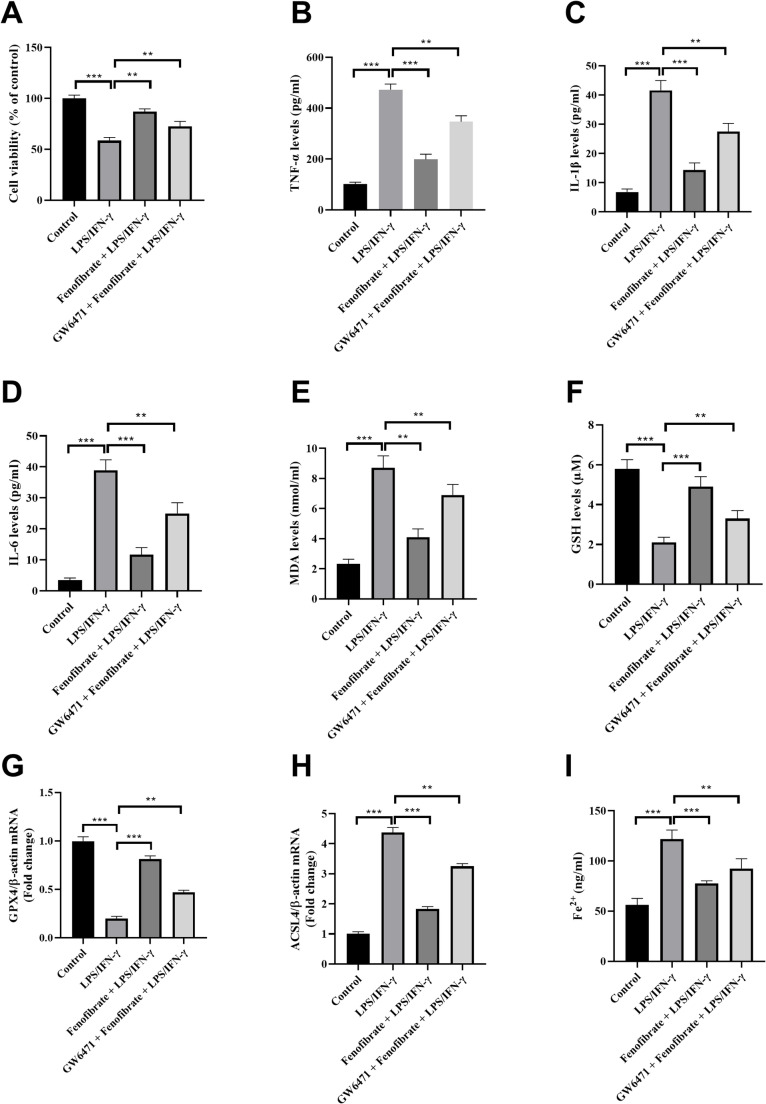
PPAR-α inhibition attenuates the protective effects of fenofibrate on inflammation and ferroptosis in activated HMC3 microglial cells. **A** CCK-8 cell viability analysis. Quantification of pro-inflammatory cytokines **B** TNF-α, **C** IL-1β, and **D** IL-6. **E** Intracellular Fe²⁺ levels and ferroptosis-related proteins **F** GPX4 and **G** ACSL4. Cells were pre-treated with the selective PPAR-α antagonist GW6471 for 1 h followed by fenofibrate (10 µM) treatment for 1 h prior to stimulation with LPS (100 ng/mL) and IFN-γ (10 ng/mL) for 24 h. Data are presented as mean ± SEM (*n* = 7 independent experiments). Statistical comparisons between multiple groups were performed using one-way ANOVA followed by Tukey’s post-hoc test. **p < 0.001, and ***p < 0.0001

CCK-8 analysis revealed that LPS/IFN-γ stimulation significantly reduced cell viability to 58.6 ± 4.2% compared with the control group (100 ± 5.1%, *p* < 0.0001, Fig. 8A). Treatment with fenofibrate markedly improved cell viability to 86.9 ± 3.7% (*p* = 0.0012 vs. LPS/IFN-γ), indicating a protective effect against inflammatory injury. However, pretreatment with GW6471 significantly attenuated the cytoprotective effect of fenofibrate, reducing cell viability to 74.6 ± 4.5% (*p* = 0.0019 vs. Fenofibrate + LPS/IFN-γ).

Consistent with these findings, LPS/IFN-γ stimulation markedly increased the production of pro-inflammatory cytokines, including TNF-α (472.5 ± 28.4 pg/ml), IL-1β (41.6 ± 4.2 pg/ml), and IL-6 (38.9 ± 3.6 pg/ml) compared with control levels (TNF-α: 102.4 ± 12.3 pg/ml; IL-1β: 6.8 ± 1.1 pg/ml; IL-6: 3.4 ± 0.9 pg/ml; all *p* < 0.0001, Fig. 8B-D). Fenofibrate treatment significantly suppressed these increases (TNF-α: 198.7 ± 21.6 pg/ml, *p* < 0.0001; IL-1β: 14.3 ± 2.5 pg/ml, *p* < 0.0001; IL-6: 11.7 ± 2.1 pg/ml, *p* < 0.0001 vs. LPS/IFN-γ). Notably, GW6471 pretreatment significantly attenuated the anti-inflammatory effects of fenofibrate (TNF-α: 346.8 ± 26.9 pg/ml, *p* = 0.0021; IL-1β: 27.5 ± 3.4 pg/ml, *p* = 0.0016; IL-6: 24.9 ± 3.1 pg/ml, *p* = 0.0024 vs. Fenofibrate + LPS/IFN-γ).

Fenofibrate also significantly reduced oxidative stress markers. LPS/IFN-γ exposure increased MDA levels to 8.7 ± 0.9 nmol/ml, whereas fenofibrate treatment significantly decreased MDA levels to 4.1 ± 0.6 nmol/ml (*p* < 0.0001, Fig. 8E). Pretreatment with GW6471 partially reversed this effect, increasing MDA levels to 6.9 ± 0.7 nmol/ml (*p* = 0.0032 vs. Fenofibrate + LPS/IFN-γ). In parallel, GSH levels were significantly depleted in LPS/IFN-γ-treated cells (2.1 ± 0.3 µM) compared with controls (5.8 ± 0.5 µM, *p* < 0.0001). Fenofibrate restored GSH levels to 4.9 ± 0.4 µM (*p* < 0.0001, Fig. 8F), whereas GW6471 pretreatment significantly reduced this protective effect (3.3 ± 0.4 µM, *p* = 0.0015 vs. Fenofibrate + LPS/IFN-γ).

Regarding ferroptosis-related markers, LPS/IFN-γ stimulation markedly decreased GPX4 levels (0.20 ± 0.03) and increased ACSL4 expression (4.38 ± 0.39) compared with the control group (GPX4: 1.00 ± 0.07; ACSL4: 1.00 ± 0.16; both *p* < 0.0001, Fig. 8G and H). Fenofibrate treatment significantly restored GPX4 levels (0.81 ± 0.06, *p* < 0.0001) and reduced ACSL4 expression (1.83 ± 0.28, *p* < 0.0001 vs. LPS/IFN-γ). In contrast, GW6471 pretreatment significantly attenuated these effects, resulting in reduced GPX4 levels (0.47 ± 0.05, *p* = 0.0011) and increased ACSL4 expression (3.25 ± 0.36, *p* = 0.0019) compared with the Fenofibrate + LPS/IFN-γ group. Similarly, intracellular Fe²⁺ levels were significantly elevated following LPS/IFN-γ stimulation (121.8 ± 9.6 ng/ml) compared with the control group (52.7 ± 4.1 ng/ml, *p* < 0.0001, Fig. 8I). Treatment with fenofibrate markedly reduced intracellular Fe²⁺ accumulation to 76.3 ± 6.2 ng/ml (*p* < 0.0001 vs. LPS/IFN-γ), indicating a strong inhibitory effect on iron-dependent oxidative damage. However, pretreatment with the PPAR-α antagonist GW6471 partially reversed this effect, increasing Fe²⁺ levels to 92.4 ± 8.7 ng/ml (*p* = 0.0026 vs. Fenofibrate + LPS/IFN-γ).

Collectively, these findings demonstrate that pharmacological inhibition of PPAR-α significantly attenuates the protective effects of fenofibrate on microglial activation, oxidative stress, and ferroptosis, suggesting that these effects are at least partially mediated through PPAR-α-dependent signaling pathways.

## Discussion

Microglial activation represents a fundamental mechanism underlying neuroinflammatory cascades and progressive neuronal injury in diverse CNS disorders. When exposed to classical inflammatory stimuli such as LPS and IFN-γ, microglia adopt an M1-like phenotype characterized by increased secretion of TNF-α, IL-1β and IL-6, overproduction of ROS, and loss of neuroprotective functions. This persistent activation state drives oxidative stress, synaptic dysfunction, and neuronal apoptosis in diseases such as AD, PD, and stroke [4, 6]. Importantly, recent research has established that metabolic reprogramming—particularly involving lipid metabolism—is a major determinant of microglial phenotype [9]. To our knowledge, this study is among the first to report that fenofibrate modulates microglial activation in association with changes in lipid metabolism and ferroptosis-related pathways. Our data demonstrate that fenofibrate suppresses inflammatory cytokine production, attenuates ROS generation, reverses LD accumulation, and restores ferroptosis-related molecular balance by downregulating ACSL4 and upregulating GPX4 in LPS/IFN-γ-activated HMC3 microglia.

Lipid metabolism serves as a critical interface between energy homeostasis, membrane remodeling, and immune signaling in microglia [8]. Under pathological stress, excessive lipid accumulation in glial cells promotes pro-inflammatory activation and functional impairment [23]. Consistent with these reports, our study demonstrated that inflammatory activation of HMC3 cells upregulated LD-associated enzymes—GPAT4, DGAT1, and PLIN2—accompanied by a marked increase in LD density. Fenofibrate treatment significantly reversed these changes, reducing LD accumulation and downregulating these lipid-metabolic regulators. Functional analyses further demonstrated that fenofibrate enhanced mitochondrial fatty acid oxidation and restored lipolytic activity, indicating a shift from lipid storage toward lipid catabolism. These findings align with studies showing that PPAR-α activation enhances β-oxidation, limits LD biogenesis, and alleviates lipotoxicity [10, 21]. Comparable results were reported in glioblastoma cells, where fenofibrate decreased DGAT1 expression and small LD formation, inducing oxidative stress and apoptosis [24]. Thus, suppression of LD synthesis and restoration of lipid turnover by fenofibrate may protect microglia from entering a dysfunctional, pro-inflammatory state that perpetuates neurodegeneration. Our data reveal that the anti-lipotoxic effect of fenofibrate is mechanistically rooted in its capacity to enhance lipid catabolism. Specifically, we found that inflammatory activation severely impaired mitochondrial fatty acid oxidation, a defect that was not only corrected but robustly enhanced by fenofibrate treatment. This is consistent with the canonical role of PPAR-α activation in promoting genes of the β-oxidation pathway. Concurrently, fenofibrate restored lipolytic function, as evidenced by normalized glycerol release, indicating accelerated breakdown of stored triglycerides. This dual action—boosting both mitochondrial oxidation of fatty acids and cytosolic lipolysis—provides a possible functional basis for the clearance of lipid droplets and the reduction in lipotoxicity, effectively reprogramming the activated microglia from a lipid-accumulating state to a lipid-catabolizing phenotype. While our data demonstrate that fenofibrate downregulates the expression of key lipid droplet-associated enzymes (GPAT4, DGAT1, PLIN2), it should be noted that protein levels do not always directly correlate with enzymatic activity. Future studies employing specific activity assays or metabolic flux analyses would provide stronger evidence for the functional inhibition of these pathways. Nevertheless, the concomitant reduction in neutral lipid content (Fig. 4C, D) and the functional enhancement of FAO and lipolysis (Fig. 4E, F) strongly support a net shift in lipid flux away from storage and towards catabolism.

Critically, this metabolic reprogramming may directly underlie the observed protection against ferroptosis. Lipid droplets store esterified PUFAs, which, upon lipolytic hydrolysis, can be incorporated into membrane phospholipids and become substrates for peroxidation. We hypothesize that the fenofibrate-induced reduction in LD biogenesis (via downregulation of PLIN2, DGAT1, GPAT4) and the concurrent enhancement of lipolysis and β-oxidation may create a metabolic shunt. This shunt redirects liberated fatty acids towards mitochondrial energy production, thereby diverting them from the ferroptotic peroxidation pathway. This model is supported by the concurrent upregulation of the protective enzyme GPX4 and aligns with studies showing that DGAT1 inhibition can sensitize cells to ferroptosis by increasing free PUFA pools, whereas enhancing β-oxidation is protective [25, 26]. Collectively, these changes effectively reprogram the activated microglia from a lipid-accumulating, ferroptosis-susceptible state to a lipid-catabolizing, protected phenotype.

Ferroptosis has emerged as a key mechanism linking lipid metabolic imbalance with microglial dysfunction [27–29]. Kapralov et al. demonstrated that “redox lipid reprogramming” in macrophages and microglia governs ferroptotic susceptibility through modulation of PUFA metabolism and 15-lipoxygenase activity [15]. Moreover, microglia under inflammatory stress display reduced antioxidant reserves, increased iron deposition, and heightened vulnerability to ferroptosis [30, 31]. Consistent with this concept, LPS/IFN-γ activation in our model significantly increased ROS, MDA, and intracellular Fe²⁺ levels while depleting GSH, collectively indicating ferroptotic stress. Fenofibrate treatment normalized these parameters, paralleling the effects of the ferroptosis inhibitor ferrostatin-1. At the molecular level, fenofibrate downregulated ACSL4 and upregulated GPX4 at both mRNA and protein levels, confirming a shift toward an anti-ferroptotic phenotype. These effects were associated with decreased Prussian Blue-positive iron deposits. Such dual regulation of ACSL4 and GPX4 mirrors observations in other ferroptosis-related contexts where suppression of ACSL4 or overexpression of GPX4 mitigates neuronal and glial cell death [22, 32]. Our experiments using the GPX4 inhibitor RSL3 provide direct functional evidence for the role of ferroptosis in microglial activation and the protective mechanism of fenofibrate (Fig. 6F, G). RSL3 alone significantly reduced cell viability, confirming that direct inhibition of GPX4 is sufficient to trigger ferroptotic death in HMC3 microglia. Importantly, when RSL3 was combined with LPS/IFN-γ stimulation, cell death was synergistically exacerbated compared to either treatment alone, demonstrating that inflammatory activation sensitizes microglia to ferroptosis. This finding aligns with the concept that pro-inflammatory polarization depletes antioxidant reserves and enriches membranes with oxidizable PUFAs, thereby lowering the threshold for ferroptotic execution [15, 27]. Fenofibrate pre-treatment robustly protected against this combined insult, restoring viability to levels comparable to RSL3 alone. Notably, fenofibrate also partially attenuated RSL3-induced cell death in the absence of inflammatory activation, suggesting that fenofibrate directly strengthens GPX4-dependent antioxidant capacity independently of its anti-inflammatory effects. The observation that the ferroptosis inhibitor Fer-1 fully rescued LPS/IFN-γ-induced viability loss, and that combining fenofibrate with Fer-1 provided no additive benefit, confirms that fenofibrate’s cytoprotective action converges on the ferroptosis pathway rather than acting through parallel, independent mechanisms. Collectively, these results suggest that fenofibrate appears to act as a metabolic and redox modulator that may protect microglia from ferroptosis by curbing lipid peroxidation and iron overload.

The interplay between PPAR-α activation and ferroptosis regulation remains mechanistically intriguing. PPAR-α can transcriptionally upregulate antioxidant and fatty acid oxidation genes such as CPT1A, ACOX1, and SOD2, thereby reducing substrate availability for lipid peroxidation [33]. Moreover, PPAR-α signaling has been reported to cross-communicate with PPAR-γ and Nrf2 pathways, amplifying antioxidant responses and anti-inflammatory signaling [34, 35]. Fenofibrate’s ability to enhance GSH levels and suppress MDA formation observed in this study may thus arise from both direct PPAR-α activation and secondary Nrf2 induction, collectively mitigating ferroptotic stress. This multidimensional regulatory axis is further supported by evidence that fenofibrate can improve redox homeostasis independently of its lipid-lowering effects [36]. Therefore, the observed upregulation of GPX4 and restoration of redox balance in HMC3 microglia may represent an outcome of PPAR-α–dependent transcriptional control. These findings suggest that fenofibrate acts as a metabolic and redox modulator, protecting microglia from ferroptosis by curbing lipid peroxidation and iron overload. Thus, targeting lipid-ferroptosis crosstalk through PPAR-α activation may represent a promising strategy for controlling microglial-driven neuroinflammation. However, preclinical validation in vivo is essential to confirm whether the molecular effects observed in HMC3 cells translate into functional neuroprotection. Furthermore, combining fenofibrate with agents that synergistically activate antioxidant defenses—such as Nrf2 inducers or ferroptosis inhibitors—may potentiate its efficacy.

Interestingly, the dual action of fenofibrate on lipid and iron homeostasis aligns with recent findings implicating metabolic-immune coupling in microglial plasticity. Metabolomic studies have revealed that activated microglia undergo a metabolic shift toward glycolysis and impaired fatty acid oxidation, resulting in iron sequestration and ROS amplification [37]. Pharmacological activation of PPAR-α or AMP-activated protein kinase (AMPK) restores oxidative metabolism and reduces neuroinflammation, indicating that metabolic rewiring may dictate inflammatory fate decisions [38]. Our results provide molecular evidence for this concept, showing that fenofibrate restores mitochondrial potential and limits Fe²⁺ accumulation in parallel with suppression of lipid peroxidation. The reduction of Prussian Blue-positive iron deposits further underscores the intimate relationship between lipid dysregulation and iron metabolism in determining microglial viability.

Fenofibrate is a clinically approved PPAR-α agonist known to stimulate fatty acid oxidation and suppress inflammatory signaling. Previous studies have reported that fenofibrate reduces glial activation, oxidative stress, and neuronal injury in models of neuropathy, PD, and neurodegeneration [39–41]. In macrophages and astrocytes, fenofibrate has been shown to inhibit NF-κB-dependent cytokine release and promote anti-inflammatory polarization [22]. Our results extend these findings to microglial cells, revealing that fenofibrate not only dampens inflammatory cytokine production but also corrects the lipid and iron imbalances that sensitize microglia to ferroptosis. This integrated effect may explain fenofibrate’s neuroprotective actions reported in other disease contexts. Mechanistically, PPAR-α activation likely orchestrates transcriptional programs that enhance lipid catabolism, reinforce antioxidant defenses, and repress genes involved in ferroptotic lipid peroxidation. To further clarify the mechanistic involvement of PPAR-α signaling in fenofibrate-mediated protection, we incorporated pharmacological inhibition experiments using the selective PPAR-α antagonist GW6471. Blocking PPAR-α signaling significantly attenuated the anti-inflammatory, antioxidant, and anti-ferroptotic effects of fenofibrate in activated HMC3 microglia. In particular, GW6471 partially reversed fenofibrate-mediated reductions in pro-inflammatory cytokine production, intracellular iron accumulation, and lipid peroxidation while diminishing the restoration of GPX4 levels. These findings strongly suggest that activation of PPAR-α plays a critical role in mediating the metabolic reprogramming and ferroptosis suppression observed in fenofibrate-treated microglia. Nevertheless, further studies involving genetic or transcriptional validation of PPAR-α target genes will be required to fully elucidate the underlying regulatory mechanisms.

In summary, this study demonstrates that fenofibrate treatment is associated with reduced microglial activation, altered lipid metabolism, and decreased ferroptosis signaling in vitro. By reducing LD accumulation (via downregulation of GPAT4, DGAT1, and PLIN2) and preventing ferroptotic signaling (via suppression of ACSL4 and induction of GPX4), fenofibrate significantly attenuates oxidative stress and pro-inflammatory cytokine production in activated microglia. These findings highlight a novel therapeutic mechanism linking lipid metabolic control with ferroptosis inhibition in microglial cells and provide a mechanistic framework for targeting lipid-ferroptosis crosstalk to mitigate microglial-driven neuroinflammation. However, this study has certain limitations that warrant consideration. A primary limitation is its reliance on an immortalized human microglial cell line. While HMC3 cells are a valuable and widely used model, they may not fully recapitulate the complex physiology, heterogeneity, and tissue context of primary microglia in vivo. To validate the translational potential of our findings, future studies should employ primary microglial cultures from rodent or human sources. Future studies should evaluate fenofibrate in vivo using relevant models of neuroinflammation and neurodegeneration to determine whether the observed modulation of lipid metabolism and ferroptosis translates into functional neuroprotection. Additionally, the relative contributions of PPAR-α–dependent versus –independent mechanisms to fenofibrate’s actions remain to be fully elucidated through genetic or pharmacological manipulation. Moreover, while our GW6471 antagonist experiments suggest PPAR-α dependency, direct measurement of PPAR-α target gene expression (e.g., CPT1A, ACOX1) was not performed, and the relative contributions of PPAR-α-dependent versus independent mechanisms remain to be fully elucidated through genetic or more comprehensive pharmacological manipulation. Despite these limitations, our in vitro findings offer a robust mechanistic foundation and justify further preclinical investigation into the repositioning of fenofibrate as a potential therapeutic agent for neuroinflammatory and neurodegenerative disorders.

## Supplementary Information

Below is the link to the electronic supplementary material.

Supplementary Material 1

## Data Availability

No datasets were generated or analysed during the current study.
