# Supplementary figures and images for: Fenofibrate mitigates microglial activation by reprogramming lipid metabolism and inhibiting ferroptosis
Source: Mol Cell Biochem. 2026 Apr 28;481(6):2529–47. doi: 10.1007/s11010-026-05544-8 (PMC13279494; doi:10.1007/s11010-026-05544-8)

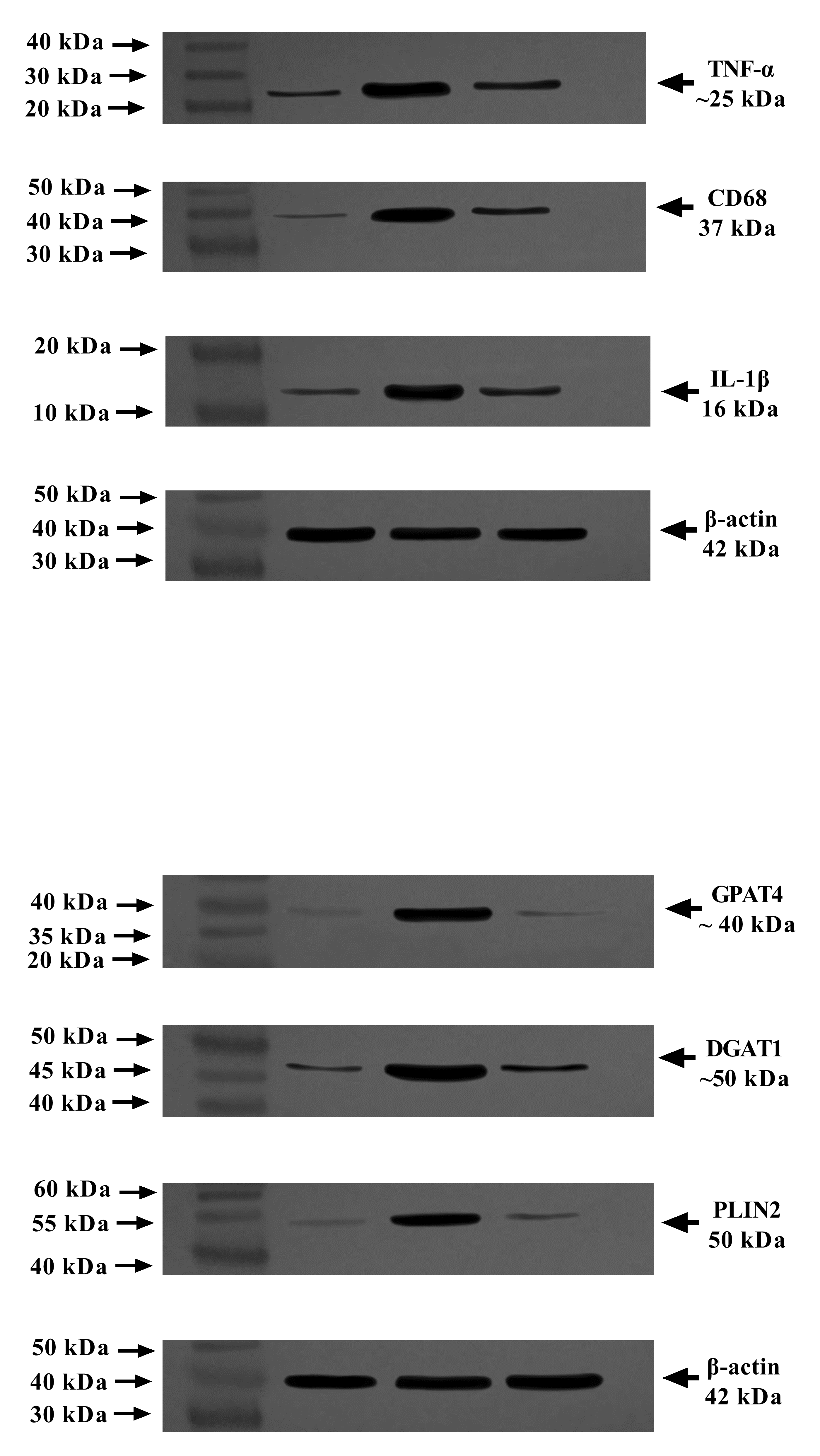

Supplement: Supplementary file 1 — Supplementary Material 1 [file 11010_2026_5544_MOESM1_ESM.tif]
